# Hypertriglyceridemia Is Associated with Reduced Leukoaraiosis Severity in Patients with a Small Vessel Stroke

**DOI:** 10.1155/2018/1361780

**Published:** 2018-08-09

**Authors:** Daqiang Ke, Feng Zhou, Hui Liang, Yang Xu, Haiyan Lou

**Affiliations:** ^1^Department of Neurology, The First Affiliated Hospital, School of Medicine, Zhejiang University, Qingchun Road 79, Hangzhou, Zhejiang 310003, China; ^2^Department of Neurology, Yijishan Hospital, Wannan Medical College, Hangzhou, Zhejiang 310003, China; ^3^Department of Radiology, The First Affiliated Hospital, School of Medicine, Zhejiang University, Hangzhou, Zhejiang 310003, China

## Abstract

Intracranial hemorrhage or microbleeds and leukoaraiosis have an overlap in biology. Hyperlipidemia may reduce the risk of ICH or cerebral microbleeds; studies focusing on the relationship between different lipid profiles and severity of periventricular hyperintensities (PVH) and subcortical white matter lesions (SWMLs) in the cerebral small vessel disease are limited. *Methods*. Patients with recent first lacunar infarct were recruited. PVH and SWMLs were accessed on MRI with the Fazekas scale, and lipid levels were measured. Univariate and multivariable regression analyses were used to assess the relation between different lipid profiles and severity of PVH and SWMLs. *Results*. In univariate analyses, advancing age was correlated with increasing severity of leukoaraiosis (*P* < 0.001). There was an inverse relationship between hypertriglyceridemia (hyper-TG) (≥1.7 mmol/l) and severity of leukoaraiosis (*P* < 0.05). In the multivariable analysis, after controlling for age, sex, and significant risk factors in the univariate and age-adjusted analyses, hyper-TG demonstrated a protective effect on the severity of PVH and SWMLs (*P* < 0.05). Higher total cholesterol (TC), high-density lipoprotein (HDL), and low-density lipoprotein (LDL) were not associated with leukoaraiosis. *Conclusions*. Hyper-TG is associated with the severity of leukoaraiosis independent of other risk factors, and it might be a protective role in cerebral small vessel disease.

## 1. Introduction

Leukoaraiosis, also known as white matter hyperintensities, is strongly linked to the cerebral small vessel disease, which increases the risk of stroke, cognitive decline, dementia, and infarct progression [1, 2]. The abnormal signals with a high intensity are often divided into 2 categories: periventricular hyperintensity (PVH), which is about the cerebral ventricles, and subcortical white matter lesions (SWMLs), which are patchy areas of white matter hyperintensities in subcortical white matter distinct from the periventricular area [3]. Although the pathogenesis of leukoaraiosis is not completely elucidated, previous studies have shown that white matter lesions are more common in ageing patients, in patients with hypertension, diabetes mellitus, and cerebral vessel atherosclerosis, and in patients with a history of stroke [1, 4].

Hyperlipidemia appears to have a different role in intracranial hemorrhage and cerebral microbleeds, although it increases the risk of coronary artery disease and ischemic stroke [5, 6]. It has been hypothesized that very low cholesterol levels may contribute to the development of a fragile endothelium, prone to leakage and rupture [7]. There has been a presumed overlap in biology between intracranial hemorrhage or microbleed and white matter hyperintensities while the relationship between hyperlipidemia and leukoaraiosis severity is still controversial [8]. Several studies have found that hyperlipidemia was independently related to decreased severity of leukoaraiosis in the elderly or patients with an ischemic stroke [9, 10]. In other studies, there were no statistical differences between hyperlipidemia and leukoaraiosis [11, 12]. Some research even found that hypertriglyceridemia (hyper-TG) was associated with increased risks of white matter lesions [13]. In these studies, PVH and SWMLs were not divided when evaluating the severity of leukoaraiosis or serum lipid fractions were not investigated. PVH have been consistently associated with different clinical, histopathological, and etiological correlates from SWMLs. The presence of aortic atherosclerosis during midlife and carotid atherosclerosis and lower hemoglobin level were preferentially associated with PVH [3, 14]. In addition, various serum lipid fractions have different roles in the intracerebral hemorrhage or cerebral microbleeds [6].

Therefore, the purpose of the present study was to investigate the relationship between lipid fractions and severity of PVH and SWMLs in the cerebral small vessel disease in Chinese patients, which had never been reported before.

## 2. Methods

### 2.1. Subjects

We selected all consecutive patients older than 18 years of age who visited from January1, 2012, to December 31, 2015, in a clinic center affiliated with the First Affiliated Hospital of Zhejiang University School of Medicine. This study was approved by the hospital's research ethics committee.

A recent first infarct was defined as a hyperintense area on diffusion imaging (with a corresponding reduced signal on apparent diffusion coefficient image processing), in a distribution compatible with an arterial territory. An experienced neurologist classified patients as having lacunar stroke according to the TOAST criteria [15]. We excluded patients with contraindications to MRI, hemorrhage, severe stroke, nonstroke diagnoses, or incomplete vascular risk factor (vascular risk factor) information. To increase the chance that the lacunar stroke results from the small vessel disease, patients with evidence of a cardiac embolic source (atrial fibrillation, myocardial infarction < 6 weeks, prosthetic cardiac valve, endocarditis, cardiomyopathy, mitral stenosis, left ventricular aneurysm, or thrombus) or cerebral large vessel disease (at least 1 internal carotid artery stenosis of 50% on MR angiography or CT angiography) were excluded.

### 2.2. Baseline Characteristics

In the study, age, sex, and vascular risk factors were recorded for each subject. Data were directly abstracted through the patient and/or proxy interview and medical chart review. Vascular risk factors were coded according to the definitions of international guidelines as follows: hypertension (systolic blood pressure > 140 mmHg and diastolic blood pressure > 90 mmHg or use of antihypertensive medication) and diabetes mellitus (self-reported disease, use of insulin or oral hypoglycemic pills, or a nonfasting plasma glucose > 11 mmol/L). Coronary artery disease was diagnosed when there was a history of angina pectoris or myocardial infarction. Patients were defined as a smoker if there was a history of cigarette smoking during the past 5 years.

### 2.3. Laboratory Methods

Blood samples were collected at enrollment in tubes containing EDTA, and the plasma levels of fasting total cholesterol(TC), triglyceride (TG), high-density lipoprotein (HDL), and low-density lipoprotein (LDL) were measured by enzymatic methods (Boehringer Mannheim, Mannheim, Germany). Routine laboratory data were analyzed for all patients within 72 hours after admission, including routine blood tests, liver and kidney function, and antiphospholipid antibodies. Serum lipid levels were dichotomized separately as two levels [16]: TC (≥5.18 mmol/l versus <5.18 mmol/l), TG (≥1.7 mmol/l versus <1.7 mmol/l), HDL (≥2.04 mmol/l versus <2.04 mmol/l), and LDL (≥3.37 mmol/l versus <3.37 mmol/l).

### 2.4. Image Acquisition and Analysis

Brain MRI studies were performed with a 1.5 T whole-body scanner (GE Signa Excite II, GE Medical Systems) or 3.0 T whole-body scanner (Achieva, Siemens Medical Systems) on all subjects. The imaging protocol consisted of diffusion-weighted imaging, apparent diffusion coefficient, T2-weighted image, T1-weighted image, and fluid-attenuated inversion recovery (FLAIR) image in the transverse plane with a slice thickness of 5 mm.

PVH and SWMLs were evaluated separately based on their distinct subcortical distributions on FLAIR sequences and classified by a neurologist and a radiologist according to the Fazekas scale, which rates lesions in both regions from 0 to 3 [17].

This study was approved by the local institutional review board/institutional ethics committee. All participants or their approved proxy provided informed consent for the participation.

### 2.5. Statistical Analysis

The Statistical Package for the Social Sciences (SPSS, ver. 15.0; SPSS Inc., Chicago, IL, USA) was used for all analyses. Chi-squared tests for dichotomous variables and ANOVA for age were used to assess their relation with different severity grades of leukoaraiosis. An ordinal logistic regression with multivariable models was fitted to describe the relationships between different severities of PVH and SWMLs from the initial MRI scan and fasting lipid profiles. Given the potent and well-established relation between advancing age and white matter hyperintensity progression, all univariate analyses were also completed after adjusting for age. Probability values were 2-tailed, and values of *P* < 0.05 were considered statistically significant.

## 3. Results

Between January 1, 2012, and December 31, 2015, a total of 1270 consecutive patients with a first ever ischemic stroke were admitted to our hospital. 396 patients were diagnosed with a lacunar stroke, and 90 patients (22.7%) with a cardiac embolic source or cerebral large vessel disease were excluded. Overall, 206 patients with a recent lacunar stroke (age: 68.2 ± 11.4 years (mean ± standard deviation)) were enrolled into the study in which 34% of the patients were female. Among them, 85% of the hypertensive patients (127/150) had taken antihypertensive medicines and 7.3% of the patients (16/206) had taken statins.

In univariate analyses (Table 1), increased age was well correlated with increasing severity of leukoaraiosis. There was an inverse relationship between hyper-TG (≥1.7 mmol/l) and severity of leukoaraiosis in both groups (*P* < 0.01). Higher TC (≥5.18 mmol/l) was inversely associated with severity of SWMLs, but not with PVH. Hypertension was only associated with severity of SWMLs. The distribution in serum lipid concentration according to the LA grade was provided in Table 2.

After adjustment for age (Table 3), higher TC, HDL, and LDL were not associated with leukoaraiosis while hyper-TG showed an inverse relationship with the severity of PVH and SWMLs (*P* < 0.001 and *P* = 0.001). In the multivariable analyses (Table 4), hyper-TG demonstrated a protective effect on the severity of PVH and SWMLs, independently of age, sex, vascular risk factors, and statin use (*P* < 0.05). There was no significant relationship between other lipid profiles and the severity of leukoaraiosis.

## 4. Discussion

In our study, we found that hyper-TG may be associated with reduced leukoaraiosis severity both in PVH and in SWML groups. This observation is consistent with prior studies where an inverse relationship between hyperlipidemia and intracranial hemorrhage risk or cerebral microbleeds was observed [5, 6]. This suggests that patients with hyper-TG appear to be at reduced risk of the cerebral small vessel disease.

The association of age with white matter hyperintensity burden was found in the study. This is consistent with prior reports [4, 9]. Hypertension has been reported as the main risk factor for small vessel diseases, and also it is related to white matter hyperintensities. We observed that hypertension increases the risk of severity of white matter hyperintensities in the SWML group, but not in the PVH group. Longitudinal investigations revealed that well-controlled blood pressure in patients with hypertension significantly reduces the risk of severe white matter hyperintensity lesions [18]. Our results from the subjects who had taken antihypertensive treatment (85%) might support this speculation.

It has been described for the first time by Jimenez-Conde et al. that a history of hyperlipidemia independently relates to decreased severity of white matter hyperintensities in patients with an ischemic stroke [9]. However, it is unclear how various serum lipid fractions were associated with the severity of white matter hyperintensities. In contrast, it has been reported that there were no statistic differences between hyperlipidemia and severity of leukoaraiosis in a few studies [4, 11, 19]. The discrepancies between ours and others may be partly explained by the following: (1) the subjects with TOAST stroke subtypes included in their studies are more than those with the cerebral small vessel disease and (2) the severity scale was not used in the classification for leukoaraiosis. One study on healthy Japanese adults showed that hyper-TG was associated with increased risks of leukoaraiosis independently of elevated blood pressure [13]. In that study, MR imaging examinations were performed using 0.4 T open MRI. The sensitivity and specificity for leukoaraiosis were lower when compared to 1.5 T or 3.0 T MR scan, which provides better spatial resolution and higher signal-to-noise ratio and contrast-to-noise ratio. Thus, the results reflecting the effect of TG on severe white matter hyperintensities in that study may not be reliable. In another study of Chinese patients, the FLAIR sequence was not used while it is more sensitive to white matter pathology than T_2_-weighted sequences [20]. FLAIR may null the signal from CSF and allow the periventricular region to be easily detected [21].

The mechanisms of the relationship between TG levels and leukoaraiosis were largely unknown. It has been estimated that up to half of the white matter is composed of myelin in the brain. A noteworthy characteristic of myelin is that it contains 70% lipids, which are approximately opposite to the situation found in most other cell membranes [22]. This could explain the role of elevated TG and CH levels in chronic cerebral injury (such as the processes involved in white matter hyperintensity development). Other possible explanations might be based on some shared genetic burden, given that both hyperlipidemia and severity of white matter hyperintensities have a significant heritability component [23]. Future studies may be needed to unveil novel clues in this field. For example, why hyper-TC is not associated with leukoaraiosis observed in our study? It is possible that the major type of dyslipidemia in China is hyper-TG rather than hyper-TC [24].

The first limitation to our study is that we only studied Chinese, and therefore our results might not necessarily apply to other ethnicities. Second, the study subjects were patients in a single medical center, and the study population may not represent the general population, which likely limits our study power. Third, only dichotomous levels were chosen to characterize the high-lipid versus low-lipid groups since the treatment was selected with the criterion of dyslipidemia according to the medical insurance system in China. Fourth, the characteristics of the research did not permit us to clearly separate the contribution of lipid-lowing treatment to the hyperlipidemia effect. However, only a small number of patients (16 patients) had taken statins before admission. In conclusion, our findings suggest that higher TG might have a protective role in leukoaraiosis, and the clinical implications of these findings warrant further investigation.

## Figures and Tables

**Table 1:** Demographic characteristics of patients according to the severity of leukoaraiosis.

	PVH					SWMLs				
	0	1	2	3	*P*	0	1	2	3	*P*
Age, y	52 (4.8)	63.2 (0.6)	71 (10.1)	75.2 (8.8)	<0.001	58.3 (9.5)	68.8 (10.7)	72.6 (9.4)	74.3 (10)	<0.001
Sex, female	21.4	37.7	32.4	34.7	0.684	24	28.8	33.3	31.6	0.768
DM	28.6	27.5	31.1	26.5	0.948	22.0	28.8	35.6	28.9	0.545
HTN	78.6	71.0	67.6	81.6	0.347	70.0	60.3	84.4	86.8	0.005
CHD	7.1	4.30	4.1	8.2	0.744	4.0	4.1	11.1	2.6	0.271
Smoking	42.9	43.5	41.9	30.6	0.511	36	41.1	46.7	34.2	0.63
Taking statins	7.1	5.8	6.8	10.9	0.738	6	6.8	4.4	15.8	0.22
Higher TG	57.1	39.1	28.4	16.3	0.008	50	30.1	24.4	15.8	0.004
Higher TC	21.4	17.4	12.2	10.2	0.555	26	9.6	15.6	5.3	0.022
Higher HDL	57.1	55.1	56.8	61.2	0.928	50	60.3	53.3	65.8	0.43
Higher LDL	14.3	14.5	17.6	12.2	0.875	24	11	15.6	10.5	0.195

Severity of leukoaraiosis is measured according to the total score on the Fazekas scale. Age is represented by the mean of years (SD) in each subgroup. The other variables are represented by the percent of individuals who qualified for this variable in each subgroup. DM: diabetes mellitus; CHD: coronary heart disease; HTN: hypertension.

**Table 2:** Distribution in serum lipid concentration according to the leukoaraiosis grade.

	PVH				SWMLs			
	0	1	2	3	0	1	2	3
TG	2.58 ± 1.19	1.84 ± 0.8	1.47 ± 0.7	1.05 ± 0.6	248 ± 0.94	1.79 ± 0.77	1.39 ± 0.5	1.03. ± 0.51
TC	4.41 ± 1.08	4.51 ± 1.15	4.57 ± 1.13	4.47 ± 1.07	4.37 ± 0.88	4.38 ± 1.15	4.56 ± 1.08	4.66 ± 1.23
HDL	1.15 ± 0.31	1.17 ± 0.38	1.1 ± 0.28	1.04 ± 0.22	1.15 ± 0.27	1.17 ± 0.37	1.14 ± 0.34	1.08 ± 0.29
LDL	2.47 ± 0.8	2.52 ± 0.86	2.56 ± 0.77	2.35 ± 0.81	2.45 ± 0.71	2.42 ± 0.85	2.53 ± 0.77	2.59 ± 0.91

**Table 3:** Associations between lipid profiles and severity of leukoaraiosis in multivariate models (adjustment for age).

	PVH			SWMLs		
	OR	95% CI	*P* value	OR	95% CI	*P* value
Age	—	—	—	—	—	—
Sex	1.06	0.53–2.1	0.877	1.39	0.71–2.73	0.338
DM	0.84	0.47–1.51	0.561	1.21	0.69–2.15	0.508
HTN	1.27	0.69–2.32	0.438	2.4	1.31–4.42	0.005
CHD	0.9	0.28–2.91	0.87	0.89	0.88–2.82	0.847
Prior stroke	6.01	2.8–12.91	<0.001	3.42	1.69–6.94	0.001
Smoking	0.62	0.32–1.18	0.144	0.9	0.48–1.68	0.731
Taking statins	0.82	0.29–2.3	0.705	0.98	0.36–2.65	0.969
TG ≥ 1.7 mmol/l versus <1.7 mmol/l	0.34	0.19–0.63	0.001	0.37	0.2–0.67	0.001
TC ≥ 5.18 mmol/l versus <5.18 mmol/l	0.42	0.14–1.21	0.109	0.45	0.16–1.3	0.139
HDL ≥ 2.04 mmol/l versus <2.04 mmol/l	1.22	0.7–2.12	0.484	1.73	1.01–3	0.05
LDL ≥ 3.37 mmol/l versus <3.37 mmol/l	1.97	0.71–5.44	0.192	1.05	0.39–2.84	0.916

OR: odds ratio.

**Table 4:** Associations between lipid profiles and severity of leukoaraiosis in multivariate models.

	PVH			SWMLs		
	OR	95% CI	*P* value	OR	95% CI	*P* value
Age	1.11	1.08–1.14	<0.001	1.1	1.06–1.13	<0.001
Sex	0.95	0.47–1.92	0.879	1.35	0.67–2.7	0.401
DM	0.84	0.46–1.54	0.565	1.29	0.71–2.32	0.401
HTN	1.09	0.58–2.04	0.795	2.32	1.24–4.34	0.009
CHD	0.6	0.17–2.09	0.419	0.58	0.17–1.93	0.373
Smoking	1.17	0.59–2.31	0.651	1.68	0.86–3.27	0.127
Taking statins	1.04	0.35–3.03	0.95	1.35	0.26–2.07	0.567
TG ≥ 1.7 mmol/l versus <1.7 mmol/l	0.49	0.26–0.93	0.029	0.49	0.48–3.7	0.027
TC ≥ 5.18 mmol/l versus <5.18 mmol/l	0.45	0.15–1.36	0.156	0.58	0.192–1.73	0.328
HDL ≥ 2.04 mmol/l versus <2.04 mmol/l	1.01	0.57–1.8	0.97	1.51	0.86–2.64	0.153
LDL ≥ 3.37 mmol/l versus <3.37 mmol/l	2.37	0.81–6.9	0.113	1.02	0.99–2.8	0.974

OR: odds ratio.

## Data Availability

Data are available in pushup2018@163.com (password: ps12345678).
